# A Comparison of Scar Infiltration, Scar Deactivation, and Standard of Care for Treatment of Chronic, Postsurgical Pain After Cesarean Section in the Primary Setting: A Comparative Effectiveness Trial

**DOI:** 10.1155/prm/3774454

**Published:** 2026-07-30

**Authors:** Andrew M. Gaillardetz, Roy M. Wagner, Pamela Hughes, David A. Moss, James D. Honeycutt, Paul F. Crawford, Mauna E. Gattenby, Stephen Cagle

**Affiliations:** ^1^ Department of Family Medicine, 375th Medical Group, Scott Air Force Base, Belleville, Illinois, USA; ^2^ Department of Family Medicine, 99th Medical Group, Nellis Air Force Base, Las Vegas, Nevada, USA, af.mil; ^3^ Department of Family Medicine, 55th Medical Group, Offutt Air Force Base, Bellevue, Nebraska, USA

## Abstract

**Introduction:**

Chronic pain following Cesarean section significantly impacts quality of life. Postcesarean pain is often treated with pharmacologic therapies; however, there are no evidence‐based guidelines available for refractory pain. The objective of this study is to determine if acupuncture and lidocaine scar infiltration improve pain.

**Methods:**

This 20‐week randomized control trial took place at two Air Force Base outpatient primary care clinics. Sixty postpartum Department of Defense beneficiaries aged 18 years or older with abdominal and/or back pain following a low transverse cesarean section, at least 3 months prior, were enrolled. Participants were randomized into one of 3 groups: (1) acupuncture scar deactivation, (2) scar infiltration with lidocaine, and (3) physical therapy (PT). The outcome measures were scar quality, as measured by the Patient and Observer Scar Assessment Scale (POSAS) and pain, as measured by the Defense and Veterans Pain Rating Scale (DVPRS) pre‐ and posttreatment, and were analyzed with random effects regression.

**Results:**

After excluding individuals with missing data, 35 (58%) were analyzed for DVPRS and 51 (85%) were analyzed for POSAS measures. Participants had a mean age of 35 years, a BMI of 30 kg/m^2^, and 70% identified as White. DVPRS declined from baseline to the last visit in the PT, scar deactivation, and scar infiltration groups by −42.7%, −54.4%, and −63.6%, respectively. The POSAS measures also declined in the PT, scar deactivation, and scar infiltration groups (patient: −57.5%, −47.7%, and −38.9%; observer: −10.9%, −34.7%, and −24.2%). The acupuncture group showed a stronger decline in the POSAS observer measure as compared to PT (interaction *β* = −0.29, *p* = 0.0217), but no differences were observed for the POSAS patient measure or DVPRS. When examining responder rates, only the POSAS observer measure was significant for a 50% reduction in the outcome, but post hoc group comparisons did not reach statistical significance in Table 5.

**Conclusion:**

This study found an improvement in the DVPRS, patient‐reported Patient and Observer Scar Assessment score, and observer‐reported Patient and Observer Scar Assessment score across all treatment groups at 20 weeks compared to the initial baseline. Compared to PT, our results suggest that acupuncture was associated with a stronger improvement in the POSAS observer measure, but no other differences between treatment conditions were observed. PT is a well‐established treatment modality but requires specialist training and frequent visits. Scar deactivation is an effective treatment but also requires specialist training. Scar infiltration with lidocaine is a procedure easily performed by most primary care physicians with limited cost. Patient‐specific factors such as time availability and provider access may best guide treatment decisions for these patients.

**Trial Registration:** ClinicalTrials.gov identifier: NCT03936309

## 1. Introduction

Multiple hypotheses for postsurgical pain include development of intraperitoneal adhesions, anterior cutaneous nerve entrapment, and disruption of the autonomic nervous system within the intracellular fluid matrix known as an “interference field” [1–5]. While there is not a clearly established pathophysiologic cause or incidence rate, studies estimate up to 23% of patients experience chronic peri‐incisional pain following Cesarean section (CS) [6]. This chronic pain is defined as pain persisting beyond the expected period of healing, approximately 3 months postpartum. A workup of chronic peri‐incisional pain after CS is extensive to rule out organic causes. There is no well‐established guideline dictating standard of care once visceral causes are excluded. Treatment can include analgesics; for refractory cases which are neuropathic in nature, tricyclic antidepressants, gabapentin, or serotonin‐norepinephrine reuptake inhibitors are used. However, if pharmacologic therapy fails, pain specialists or surgical evaluation is considered.

Acupuncture in military healthcare is increasingly utilized for chronic pain. Scar deactivation, an acupuncture technique, is used for treatment of various types of scarassociated pain. Acupuncture has been used to treat scar‐associated pain following CS [7]. In theory, needle insertion into connective tissues produces analgesia through disruption and remodeling of the extracellular matrix in loose connective tissues and alters gene expression, affecting neurotransmitter levels and cell signaling pathways in fibroblasts and mast cells [8]. In traditional Chinese medicine, Qi energy flows in balanced channels throughout the healthy body. However, in areas of injury leading to scar formation, Qi stagnates and disrupts its flow, thereby causing abnormal skin sensations like pain, itching, or numbness.

Scar infiltration with lidocaine is the other modality used in many clinical settings. The anti‐inflammatory effects of local anesthetics theoretically mitigate the autonomic nervous system dysfunction of “interference fields” caused by scar tissue [9–11]. Local anesthetics promote anti‐inflammatory activity through a variety of biochemical signaling 5 mechanisms. Lidocaine injection purportedly alleviates nerve entrapment within fascia through hydrodissection, a technique effectively used for conditions such as carpal tunnel syndrome, cubital tunnel syndrome, and radial nerve entrapment [12]. However, there is limited clinical trial evidence supporting its effectiveness in the setting of chronic postsurgical pain.

Current postpartum peri‐incisional chronic pain control strategies involve pharmacotherapy and physical therapy (PT). PT has demonstrated some improvement in pain, pigmentation, thickness, and surface area of the scar [13]. Despite the existence of diverse methods to treat pain, there has been little research to evaluate the effectiveness of integrative strategies targeting the sources of postsurgical pain.

The objective of this study was to measure the effectiveness of scar infiltration with 0.5%–1% lidocaine and the effectiveness of scar deactivation with acupuncture surface release technique in reducing chronic postsurgical pain related to incision site after low transverse CS when compared to each other and standard care of PT.

## 2. Materials and Methods

### 2.1. Design and Setting

The study was an unblinded randomized controlled trial where participants were assigned to either scar deactivation, scar infiltration, or standard of care with PT. This study was approved by the Wilford Hall Ambulatory Surgical Center Institutional Review Board (FWH20190005H). Every subject signed informed consent. This study adhered to CONSORT guidelines.

Dissimilar treatment protocols by allocation group made blinding impractical. An a priori power analysis was conducted using G∗Power version 3.1.9.2 [Faul et al., 2009]. Based on published Patient and Observer Scar Assessment Scale (POSAS) total scores for nonhypertrophic scars and Defense and Veterans Pain Rating Scale (DVPRS) pain intensity values, a sample size of 45 participants was estimated to provide 95% power at an α level of 0.05 for detecting within‐group changes over time. This study ultimately recruited 60 subjects at Scott and Nellis Air Force Bases over 5 years to account for potential attrition. Subjects were scheduled for a visit every 4 weeks for 20 weeks.

### 2.2. Participants

Female active duty and Department of Defense (DoD) beneficiaries at Scott and Nellis Air Force Bases who were age 18 or older with chronic abdominal and/or back pain following a low transverse CS were eligible for the study. Pain must have persisted for 3 months or more after the CS. If participants had prior scar deactivation with surface release technique for CS scar, they must have completed a 12 week or more washout period. Exclusion criteria included: (1) currently pregnant, (2) prior scar deactivation within the last 12 weeks, (3) prior scar infiltration with lidocaine for CS, (4) active cellulitis of scar area, (5) CS scar revision, and (6) vertical incision or emergent CS.

### 2.3. Interventions

Participants were randomized into one of three groups using block randomization: (1) scar deactivation surface release technique, (2) scar infiltration with lidocaine, and (3) standard of care PT performed using the McKenzie method. For those assigned to acupuncture, a certified physician acupuncturist (a qualified physician who completed an additional 200‐ or 300‐h acupuncture course which included training in this technique) placed 40 mm needles at approximately 30–45° angles to skin in alternating fashion 1–1.5 cm apart to surround the scar. Needles were not placed at specifically defined acupuncture points but at targeted superficial fascia depth. A maximum of 20 needles were used and left in place for 20 min per treatment. For those assigned to lidocaine injection, a volume of between 30 and 60 cc of 0.51% lidocaine (3 mg/kg dose) was injected with a 1.5 inch 25G needle throughout the dermal and subcutaneous layers of scar tissue. The third group was assigned to PT, and patients were referred to in‐network physical therapists with the consult requesting the McKenzie protocol treatment. The McKenzie protocol is a standard modality of PT in which the therapist tries to find a cause‐and‐effect relationship between body positions, movement, and pain response [14]. That information is used to develop an exercise program to improve the pain.

### 2.4. Outcomes

Outcome measures were the POSAS patient, the POSAS observer, and the DVPRS. The POSAS is a validated scar assessment instrument consisting of a patient scale and an observer scale, each comprising six items rated on a 1‐10 Likert scale with a total score ranging from 6–60 (higher scores indicate worse scar quality). The instrument has demonstrated good internal consistency and inter‐rater reliability (https://pubmed.ncbi.nlm.nih.gov/15253184/, https://pubmed.ncbi.nlm.nih.gov/16079683/). The DVPRS is a validated single‐item numerical pain rating scale (0–10) developed specifically for use in military and veteran populations, with demonstrated test–retest reliability and concurrent validity with established pain measures (https://pubmed.ncbi.nlm.nih.gov/23137169/). At each visit, participants were asked to complete the DVPRS and POSAS before and after treatment.

### 2.5. Statistical Analysis

The analysis followed a modified intention‐to‐treat approach, including all randomized participants who had a baseline outcome observation and at least one postbaseline outcome observation; those missing baseline data or with no follow‐up data were excluded, as they provided no information for estimating change over time.

**FIGURE 1:**
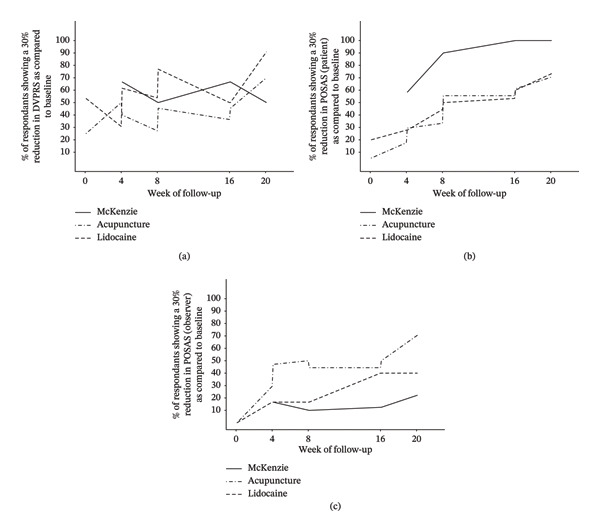
(a) Responder rate (30% reduction) in DVPRS by week of follow‐up and treatment group. (b). Responder rate (30% reduction) in POSAS (patient) by week of follow‐up and treatment group. (c) Responder rate (30% reduction) in POSAS (observer) by week of follow‐up and treatment group.

**FIGURE 2:**
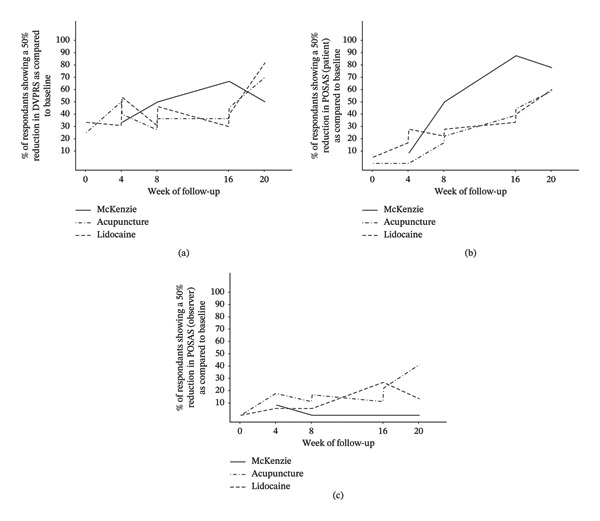
(a) Responder rate (50% reduction) in DVPRS by week of follow‐up and treatment group. (b). Responder rate (50% reduction) in POSAS (patient) by week of follow‐up and treatment group. (c) Responder rate (50% reduction) in POSAS (observer) by week of follow‐up and treatment group.

Baseline demographics (race/ethnicity, facility, and age) and outcomes (DVPRS, POSAS patient, and POSAS observer) were compared by treatment condition to verify that randomization provided comparable groups, using chi‐square tests for categorical variables and ANOVA for continuous variables. Patients who were missing data at the baseline/pretreatment time point and those who did not have any follow‐ups were excluded from the analysis. For those with available data, the association between exclusion and demographics, baseline outcome value, and treatment group was analyzed to assess potential bias using chi‐square tests for categorical variables and ANOVA for continuous variables. For those included in the final analysis, means and standard deviations (SD) were described by treatment group and time point, using outcome measures prior to treatment administration at each respective visit. Plots of mean outcomes were also generated, which included mean outcomes for each visit both before and after the visit treatment was administered. Responder rates were also calculated and reported for each outcome by time point and treatment group (Figures 1a–c and 2a–c). Responder rates were defined as a 30% or 50% reduction (both levels were assessed) in the outcome measure at follow‐up as compared to baseline, using the measures taken before treatment at each visit. The 30% and 50% thresholds are based on established standards in pain research. IMMPACT guidelines define a ≥ 30% reduction as a moderate clinically meaningful improvement and a ≥ 50% reduction as a substantial improvement [15]. Differences in responder rates at the final follow‐up (20 weeks) were analyzed using chi‐square analyses to test for overall differences, and Bonferroni‐adjusted *p* values were reported for post hoc testing to compare the Scar Deactivation and Scar Infiltration treatment groups to the PT group.

Differential changes in DVPRS and POSAS outcomes over time (pretreatment values at each visit) were analyzed using multivariable random effects regression models with random intercepts to account repeated measures. Models included a term for the treatment group, weeks postbaseline, and an interaction between those variables. The interaction term directly tests differential changes in outcomes by treatment group over time. Models were adjusted for age, race/ethnicity, and baseline BMI as covariates.

## 3. Results

**FIGURE 3:**
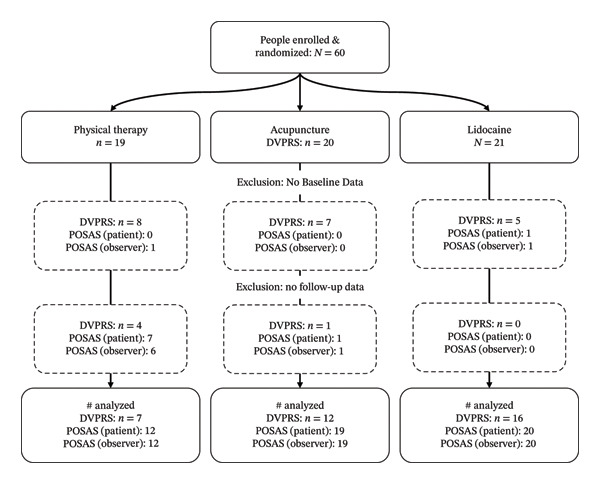
Sample randomization and exclusions based on missing data and/or losses to follow‐up.

Of the 60 people enrolled in the study, 19 were assigned to the comparison group (PT), 20 were assigned to scar deactivation, and 21 were assigned to scar infiltration (Figure 3). After excluding individuals with missing baseline outcome data and those with no followup outcome data, 35 (58%) were analyzed for DVPRS and 51 (85%) were analyzed for POSAS measures (see Supporting Information). With respect to randomization to treatment groups, we observed no statistically significant associations between treatment group and demographic variables, facility, or baseline outcome measures (all *p* > 0.05, Table 1).

**TABLE 1:** Sample description overall and comparison of attributes by treatment group, *N* = 51 participants.

Attribute	Category value	Total	McKenzie	Acupuncture	Lidocaine	*p* value
Race/Ethnicity, *n* (col %)	Asian	2 (3.9%)	0 (0.0%)	1 (5.3%)	1 (5.0%)	0.5985
	Black	5 (9.8%)	0 (0.0%)	3 (15.8%)	2 (10.0%)	0.5985
	Hispanic, Latin, or Mediterranean	4 (7.8%)	1 (8.3%)	2 (10.5%)	1 (5.0%)	0.5985
	Other, undefined or more than one	3 (5.9%)	2 (16.7%)	0 (0.0%)	1 (5.0%)	0.5985
	Pacific Islander/American Indian/Alaskan Native	1 (2.0%)	0 (0.0%)	1 (5.3%)	0 (0.0%)	0.5985
	White	36 (70.6%)	9 (75.0%)	12 (63.2%)	15 (75.0%)	0.5985

Facility, *n* (col %)	Nellis	24 (47.1%)	6 (50.0%)	9 (47.4%)	9 (45.0%)	0.9625
	Scott	27 (52.9%)	6 (50.0%)	10 (52.6%)	11 (55.0%)	0.9625

Age, mean (SD)		34.9 (7.0)	34.1 (6.0)	35.5 (6.2)	34.8 (8.6)	0.8692

BMI, mean (SD)		30.1 (5.3)	30.9 (4.2)	28.9 (4.0)	30.8 (6.7)	0.4763

Baseline DVPRS, mean (SD)		3.9 (2.3)	3.1 (2.1)	4.2 (2.1)	4.1 (2.5)	0.5916

Baseline POSAS Patient, mean (SD)		31.4 (11.5)	28.2 (12.1)	32.2 (10.3)	32.6 (12.4)	0.5581

Baseline POSAS Observer, mean (SD)		18.8 (6.0)	16.1 (5.2)	21.2 (6.8)	18.2 (5.0)	0.0574

*Note:* This analysis includes all participants included in any of the three outcome models. Statistical comparisons conducted using chi‐square for categorical variables and ANOVA for continuous variables.

**FIGURE 4:**
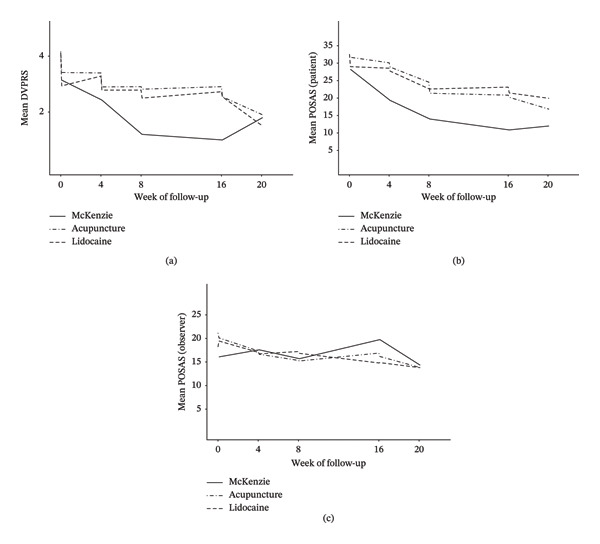
(a) DVPRS by week of follow‐up and treatment group. (b) POSAS (Patient) by week of follow‐up and treatment group. (c) POSAS (Observer) by week of follow‐up and treatment group.

DVPRS declined from baseline to the last visit in the PT, scar deactivation, and scar infiltration groups by −42.7%, −54.4%, and −63.6%, respectively (Table 2). The POSAS measures also declined in the PT, scar deactivation, and scar infiltration groups (patient: −57.5%, −47.7%, and −38.9%; observer: −10.9%, −34.7%, and −24.2%). Figures 4a–c show changes in DVPRS and both POSAS measures by week of follow‐up. DVPRS and POSAS patients generally showed declines across treatment groups.

**TABLE 2:** Mean (SD) outcomes at each follow‐up by treatment group.

Treatment group	Outcome measure	Baseline	4 Weeks	% Change from baseline, Week 4	8 Weeks	% Change from baseline, Week 8	16 Weeks	% Change from baseline, Week 16	20 Weeks	% Change from baseline, Week 20
McKenzie	DVPRS	3.1 (2.1)	2.4 (2.3)	−22.7%	1.2 (1.8)	−61.8%	1.0 (1.2)	−68.2%	1.8 (2.5)	−42.7%
Acupuncture	DVPRS	4.2 (2.1)	3.4 (2.1)	−18.4%	2.9 (1.9)	−30.2%	2.9 (1.2)	−30.2%	1.9 (1.8)	−54.4%
Lidocaine	DVPRS	4.1 (2.5)	3.3 (2.2)	−20.3%	2.8 (2.0)	−32.5%	2.7 (2.7)	−33.9%	1.5 (1.7)	−63.6%
McKenzie	POSAS (Patient)	28.2 (12.1)	19.3 (8.6)	−31.6%	14.0 (7.0)	−50.4%	10.9 (4.2)	−61.5%	12.0 (5.8)	−57.5%
Acupuncture	POSAS (Patient)	32.2 (10.3)	30.1 (11.2)	−6.3%	24.6 (8.1)	−23.6%	20.8 (10.4)	−35.2%	16.8 (9.1)	−47.7%
Lidocaine	POSAS (Patient)	32.6 (12.4)	28.6 (15.5)	−12.4%	22.7 (15.3)	−30.5%	23.1 (18.2)	−29.0%	19.9 (16.8)	−38.9%
McKenzie	POSAS (Observer)	16.1 (5.2)	17.6 (8.1)	9.3%	15.7 (5.2)	−2.4%	19.8 (9.3)	22.8%	14.3 (2.6)	−10.9%
Acupuncture	POSAS (Observer)	21.2 (6.8)	17.4 (7.4)	−18.0%	15.3 (5.3)	−27.5%	16.9 (6.9)	−20.2%	13.8 (7.2)	−34.7%
Lidocaine	POSAS (Observer)	18.2 (5.0)	17.1 (6.9)	−6.3%	17.2 (6.1)	−5.4%	14.8 (7.4)	−18.7%	13.8 (6.0)	−24.2%

*Note:* This analysis summarizes outcomes for each of the three outcomes among those included in the longitudinal analysis (DVPRS: *N* = 35; POSAS Patient: *N* = 51; POSAS Observer: *N* = 51).

Model outcomes are shown in Table 3. After adjusting for age, race/ethnicity, and BMI, scar deactivation was associated with a stronger decline in POSAS (observer) as compared to PT (interaction regression coefficient = −0.29, 95% CI ‐0.54 to −0.04, *p* = 0.0217). Although the point estimate for scar infiltration was also negative, change over time as compared to PT was not significant (interaction regression coefficient = −0.20, 95% CI ‐0.45 to 0.05, *p* = 0.1171). No significant interactions were observed for scar deactivation and scar infiltration in models examining DVPRS and POSAS patient.

**TABLE 3:** Comparison of changes in outcomes over time by treatment group.

Outcome	Independent variable	Regression coefficient (95% CI)	*p* value
DVPRS	Treatment Group: Acupuncture (ref = McKenzie)	1.58 (−0.61, 3.76)	0.1511
	Treatment Group: Lidocaine (ref = McKenzie)	1.48 (−0.48, 3.44)	0.1344
	Week of follow‐up	−0.06 (−0.14, 0.01)	0.0904
	Interaction: Week×Acupuncture	−0.03 (−0.12, 0.06)	0.4580
	Interaction: Week×Lidocaine	−0.03 (−0.12, 0.05)	0.4408

POSAS Patient	Treatment Group: Acupuncture (ref = McKenzie)	5.36 (−3.36, 14.08)	0.2229
	Treatment Group: Lidocaine (ref = McKenzie)	5.63 (−2.54, 13.80)	0.1724
	Week of follow‐up	−0.70 (−0.95, −0.45)	< 0.0001
	Interaction: Week×Acupuncture	−0.05 (−0.36, 0.26)	0.7579
	Interaction: Week×Lidocaine	0.14 (−0.17, 0.46)	0.3699

POSAS Observer	Treatment Group: Acupuncture (ref = McKenzie)	2.72 (−1.57, 7.01)	0.2100
	Treatment Group: Lidocaine (ref = McKenzie)	0.98 (−3.07, 5.04)	0.6307
	Week of follow‐up	0.01 (−0.19, 0.21)	0.9491
	Interaction: Week×Acupuncture	−0.29 (−0.54, −0.04)	0.0217
	Interaction: Week×Lidocaine	−0.20 (−0.45, 0.05)	0.1171

*Note:* Results from a random intercepts model to adjust for repeated measures, adjusted for age, race/ethnicity, and BMI at baseline. The interaction between time and treatment condition assesses whether there were differential changes over visits. Estimates for time represent changes from one visit to the next. The number of patients included in the analysis varied by outcome (DVPRS: *N* = 35; POSAS Patient: *N* = 51; POSAS Observer: *N* = 51).

## 4. Discussion

This unblinded randomized control trial investigated the effectiveness of scar deactivation with acupuncture and scar infiltration with lidocaine compared to PT (McKenzie method) for reduction in chronic peri‐incisional pain from CSs. Currently, there is no established guideline on treatment once visceral etiologies have been excluded. Scar deactivation with acupuncture and/or scar infiltration with lidocaine offer a nonsystemic, nonpharmacologic treatment option with relatively low adverse effects, especially when compared to typical pharmacologic and surgical alternatives.

This study found that, after adjusting for age, race/ethnicity, and BMI, scar deactivation with acupuncture was associated with a steeper decline in observer rated POSAS scores as compared to PT. There was also a greater decline in the scar infiltration group compared to PT with regards to observer rated POSAS scores, but these findings were not significant. There were no significant differences observed between scar deactivation with acupuncture and scar infiltration groups. It is important to note that there was an improvement in DVPRS, patient reported POSAS, and observer reported POSAS across all three treatment groups at the end of the 20‐week study compared to initial baseline, and at the conclusion of the study there were no significant difference amongst treatment groups. Age, race/ethnicity, and baseline BMI were included as covariates in the multivariable models (Section 2.5 and Table 3). Pain characteristics captured in this study (DVPRS and POSAS) are assessed as outcomes rather than covariates, and other potential confounders such as medication use were not captured in a manner suitable for adjustment. Randomization should provide balance on baseline characteristics in expectation, and Table 1 confirms no statistically significant baseline differences across groups for the variables that were measured. For the scar deactivation and scar infiltration arms, treatments were administered at study visits, so adherence is reflected in visit attendance (Table 4). For the PT arm, in‐network PT sessions occurred outside study visits, and session‐level attendance was not tracked (see Table 5).

**TABLE 4:** Comparison of attributes by exclusion from analysis, *N* = 60 participants.

	DVPRS: Included	DVPRS: Excluded	DVPRS: *p* value	POSAS Patient: Included	POSAS Patient: Excluded	POSAS Patient: *p* value	POSAS Observer: Included	POSAS Observer: Excluded	POSAS Observer: *p* value
Treatment Group, *n* (row %)									
McKenzie	7 (36.8%)	12 (63.2%)		12 (63.2%)	7 (36.8%)		12 (63.2%)	7 (36.8%)	
Acupuncture	12 (60.0%)	8 (40.0%)	0.0410	19 (95.0%)	1 (5.0%)	0.0055	19 (95.0%)	1 (5.0%)	0.0055
Lidocaine	16 (76.2%)	5 (23.8%)		20 (95.2%)	1 (4.8%)		20 (95.2%)	1 (4.8%)	
Race/Ethnicity, *n* (row %)									
Asian	2 (50.0%)	2 (50.0%)		2 (50.0%)	2 (50.0%)		2 (50.0%)	2 (50.0%)	
Black	4 (44.4%)	5 (55.6%)		5 (55.6%)	4 (44.4%)		5 (55.6%)	4 (44.4%)	
Hispanic, Latin, or Mediterranean	2 (50.0%)	2 (50.0%)		4 (100.0%)	0		4 (100.0%)	0	
Other, undefined or more than one	1 (25.0%)	3 (75.0%)	0.4995	3 (75.0%)	1 (25.0%)	0.0157	3 (75.0%)	1 (25.0%)	0.0157
Pacific Islander/American Indian/Alaskan Native	1 (100.0%)	0		1 (100.0%)	0		1 (100.0%)	0	
White	25 (65.8%)	13 (34.2%)		36 (94.7%)	2 (5.3%)		36 (94.7%)	2 (5.3%)	
Facility, *n* (row %)									
Nellis	18 (64.3%)	10 (35.7%)	0.5403	24 (85.7%)	4 (14.3%)	1.0000	24 (85.7%)	4 (14.3%)	1.0000
Scott	17 (53.1%)	15 (46.9%)		27 (84.4%)	5 (15.6%)		27 (84.4%)	5 (15.6%)	
Age, mean (SD)	34.2 (6.1)	35.7 (7.8)	0.4049	34.9 (7.0)	34.3 (6.0)	0.8267	34.9 (7.0)	34.3 (6.0)	0.8267
Body mass index, mean (SD)	29.0 (4.4)	30.8 (5.9)	0.1753	30.1 (5.3)	27.6 (3.6)	0.1749	30.1 (5.3)	27.6 (3.6)	0.1749
DVPRS and POSAS, mean (SD)	3.9 (2.3)	3.0 (0.7)	0.3651	31.4 (11.5)	23.9 (9.0)	0.0828	18.8 (6.0)	15.1 (6.0)	0.1354
Total *n* (row %)	35 (58.3%)	25 (41.7%)		51 (85.0%)	9 (15.0%)		51 (85.0%)	9 (15.0%)	

*Note:* “Included” refers to patients included in the analysis and “Excluded” refers to patients excluded due to not having baseline data or not having any follow‐up data for the outcome. Statistical comparisons conducted using chi‐square for categorical variables and ANOVA for continuous variables.

**TABLE 5:** Responder rates at 30% and 50% over time by treatment group.

Outcome variable	Treatment group	4 Weeks	8 Weeks	16 Weeks	20 Weeks	Responder rate at 20 weeks, value	Responder rate at 20 weeks, post hoc *p* value
Responder based on 30% reduction in outcome at follow‐up as compared to baseline							
DVPRS	McKenzie	4/6 (66.7%)	2/4 (50.0%)	2/3 (66.7%)	2/4 (50.0%)	0.2209	Reference
	Acupuncture	5/10 (50.0%)	3/11 (27.3%)	4/11 (36.4%)	7/10 (70.0%)		1.0000
	Lidocaine	4/13 (30.8%)	7/13 (53.8%)	5/10 (50.0%)	10/11 (90.9%)		0.6138

POSAS (Observer)	McKenzie	2/12 (16.7%)	1/10 (10.0%)	1/8 (12.5%)	2/9 (22.2%)	0.0442	Reference
	Acupuncture	5/17 (29.4%)	9/18 (50.0%)	8/18 (44.4%)	12/17 (70.6%)		0.1047
	Lidocaine	3/18 (16.7%)	3/18 (16.7%)	6/15 (40.0%)	6/15 (40.0%)		1.0000

POSAS (Patient)	McKenzie	7/12 (58.3%)	9/10 (90.0%)	8/8 (100.0%)	9/9 (100.0%)	0.1942	Reference
	Acupuncture	3/17 (17.6%)	6/18 (33.3%)	10/18 (55.6%)	12/17 (70.6%)		0.3959
	Lidocaine	5/18 (27.8%)	8/18 (44.4%)	8/15 (53.3%)	11/15 (73.3%)		0.5158

Responder based on 50% reduction in outcome at follow‐up as compared to baseline							

DVPRS	McKenzie	2/6 (33.3%)	2/4 (50.0%)	2/3 (66.7%)	2/4 (50.0%)	0.4709	Reference
	Acupuncture	5/10 (50.0%)	3/11 (27.3%)	4/11 (36.4%)	7/10 (70.0%)		1.0000
	Lidocaine	4/13 (30.8%)	4/13 (30.8%)	3/10 (30.0%)	9/11 (81.8%)		1.0000

POSAS (Observer)	McKenzie	1/12 (8.3%)	0/10 (0.0%)	0/8 (0.0%)	0/9 (0.0%)	0.0326	Reference
	Acupuncture	3/17 (17.6%)	2/18 (11.1%)	2/18 (11.1%)	7/17 (41.2%)		0.1478
	Lidocaine	1/18 (5.6%)	1/18 (5.6%)	4/15 (26.7%)	2/15 (13.3%)		1.0000

POSAS (Patient)	McKenzie	1/12 (8.3%)	5/10 (50.0%)	7/8 (87.5%)	7/9 (77.8%)	0.5975	Reference
	Acupuncture	0/17 (0.0%)	3/18 (16.7%)	7/18 (38.9%)	10/17 (58.8%)		1.0000
	Lidocaine	3/18 (16.7%)	4/18 (22.2%)	5/15 (33.3%)	9/15 (60.0%)		1.0000

*Note:* Responder rates were defined as reporting a 30% or 50% reduction in the outcome as compared to baseline. Statistical testing using chi‐square tests, with Bonferroni adjustment for post hoc tests.

These findings provide helpful guidance for real‐world applications and clinical decision‐making for those caring for patients with chronic peri‐incisional pain following CS. Both scar deactivation with acupuncture and scar infiltration with lidocaine were as effective as PT in this study, while requiring fewer visits and less overall time commitment. Reduced treatment burden has been shown to improve adherence and decrease healthcare utilization and costs, supporting the potential practical advantages of these approaches [16]. Additionally, scar infiltration with lidocaine may offer a more accessible treatment option in some settings, as it does not require referral for specialized therapies such as PT or acupuncture, potentially expanding access to care. No clinically significant adverse events were reported among any treatment group.

### 4.1. Limitations

This study does have several limitations. At the initiation of the study a power analysis was performed that found that at least 45 study participants were needed to show true statistical significance difference in results; however, due to missing patient data only 35 patients were able to be analyzed for DVPRS results. There was a reported decrease in DVPRS results from baseline for all three treatment groups, but it was not significant given that, in regard to DVPRS, this study is underpowered. A statistical difference between groups could exist but was missed due to low sample sizing. Looking at Table 2, mean results were collected for all subgroups; however, all have relatively large SD, and specifically for the PT group, the SD for DVPRS at 8, 16, and 20 weeks is larger than the reported mean. The abnormally high SD is likely due to low sample sizing and numerous outliers. In this study, the mean was used over the median to avoid affecting statistical power, but in larger studies with more patients, using median scores may present more clinically relevant data by minimizing the effects of outliers.

Another limitation is that this study had a significant data exclusion rate in the PT treatment group compared to the other two treatment groups, 12 out of 19 (63.2%) were excluded from DVPRS and 7 out of 19 (36.8%) were excluded from both patient and observer reported POSAS. The PT provided was standardized to the McKenzie method; however, the lack of complete data for the PT group, given that these patients were referred to “innetwork” physical therapists leads to some concern about the homogeneity of treatment protocols. There is also concern for possible inter‐rater variability for observer‐rated POSAS scores as a source of error. This lack of data could have significantly changed study outcomes. Regarding race/ethnicity, there was a significant data exclusion rate for patient‐ and observer‐reported POSAS scores in Asian patients compared to other subgroups.

## 5. Conclusions

There is a lack of established guidance for treating chronic peri‐incisional pain post‐CS that is refractory to pharmacologic therapy. This medical gap greatly affects patients, with upwards of 18% of postpartum women who undergo CS meeting criteria. This study showed that PT, scar deactivation with acupuncture, and scar infiltration demonstrated within‐group improvements in DVPRS and POSAS scores over 20 weeks. Although acupuncture showed a steeper decline in observer POSAS scores compared to PT, no statistical significant differences were observed between groups at the final follow‐up, and the null hypothesis was not rejected. These findings suggest that multiple treatment approaches may be reasonable to consider, and decisions can be guided by local availability, provider expertise, and patient preference. This study shows that nonpharmacological treatments can be used as treatment options and should be considered in this patient population. There were no true adverse events reported throughout this study other than one patient with temporary worsening of pain during scar deactivation which resolved by the end of the procedure. This study also shows that there is a need for larger‐scale studies to help extrapolate the benefit of the treatment groups further, as well as a need for comparing these treatment groups with systemic pharmacologic interventions.

## Author Contributions

All authors agree to be accountable for the research presented.

## Funding

There is no funding to report.

## Conflicts of Interest

The authors declare no conflicts of interest.

## Supporting Information

Additional supporting information can be found online in the Supporting Information section.

## Supporting information


**Supporting Information** A table reporting number of follow‐up data collection time points by treatment group and outcome.

## Data Availability

The data that support the findings of this study are available from the authors with completion of a Data Sharing Agreement with the Defense Health Agency. Restrictions apply to the availability of these data which are the property of the Department of Defense.
