# Supplementary material for: A Comparison of Scar Infiltration, Scar Deactivation, and Standard of Care for Treatment of Chronic, Postsurgical Pain After Cesarean Section in the Primary Setting: A Comparative Effectiveness Trial
Source: Pain Res Manag. 2026 Jul 30;2026:3774454. doi: 10.1155/prm/3774454 (PMC13422640; doi:10.1155/prm/3774454)
Supplement: Supplementary file 1 — Supporting Information A table reporting number of follow‐up data collection time points by treatment group and outcome. [file PRM-2026-3774454-s001.docx]

## Supplemental Table. Number of follow-up data collection time points by treatment group and outcome

| **Treatment group** | **Data collection DVPRS time points** | | **POSAS**  **(patient)** | **POSAS**  **(observer)** |
| --- | --- | --- | --- | --- |
| **McKenzie** | 2 | 1 (14.3%) | 2 (16.7%) | 2 (16.7%) |
| **McKenzie** | 3 | 1 (14.3%) | 0 | 0 |
| **McKenzie** | 4 | 2 (28.6%) | 3 (25.0%) | 3 (25.0%) |
| **McKenzie** | 5 | 3 (42.9%) | 7 (58.3%) | 7 (58.3%) |
| **Acupuncture** | 2 | 1 (8.3%) | 1 (5.3%) | 1 (5.3%) |
| **Acupuncture** | 4 | 3 (25.0%) | 3 (15.8%) | 3 (15.8%) |
| **Acupuncture** | 5 | 8 (66.7%) | 15 (78.9%) | 15 (78.9%) |
| **Lidocaine** | 2 | 2 (12.5%) | 2 (10.0%) | 2 (10.0%) |
| **Lidocaine** | 3 | 3 (18.8%) | 3 (15.0%) | 3 (15.0%) |
| **Lidocaine** | 4 | 1 (6.2%) | 2 (10.0%) | 2 (10.0%) |
| **Lidocaine** | 5 | 10 (62.5%) | 13 (65.0%) | 13 (65.0%) |
